# A Review on Biomedical Application of Polysaccharide-Based Hydrogels with a Focus on Drug Delivery Systems

**DOI:** 10.3390/polym14245432

**Published:** 2022-12-12

**Authors:** Bahareh Farasati Far, Mohammad Reza Naimi-Jamal, Maryam Safaei, Kimia Zarei, Marzieh Moradi, Hamed Yazdani Nezhad

**Affiliations:** 1 Research Laboratory of Green Organic Synthesis and Polymers, Department of Chemistry, Iran University of Science and Technology, Tehran 1684613114, Iran; 2 Department of Pharmacology, Faculty of Pharmacy, Eastern Mediterranean University, Famagusta 99628, Turkey; 3 Faculty of Pharmacy and Pharmaceutical Sciences, Tehran Medical Sciences, Islamic Azad University, Tehran 1916893813, Iran; 4 Faculty of Pharmacy, Shahid Beheshti University of Medical Sciences, Tehran 1985717443, Iran; 5 Department of Mechanical Engineering & Aeronautics, City University of London, London EC1V 0HB, UK

**Keywords:** polysaccharide, drug delivery, hydrogel, bioengineering, biomedical application

## Abstract

Over the last years of research on drug delivery systems (DDSs), natural polymer-based hydrogels have shown many scientific advances due to their intrinsic properties and a wide variety of potential applications. While drug efficacy and cytotoxicity play a key role, adopting a proper DDS is crucial to preserve the drug along the route of administration and possess desired therapeutic effect at the targeted site. Thus, drug delivery technology can be used to overcome the difficulties of maintaining drugs at a physiologically related serum concentration for prolonged periods. Due to their outstanding biocompatibility, polysaccharides have been thoroughly researched as a biological material for DDS advancement. To formulate a modified DDS, polysaccharides can cross-link with different molecules, resulting in hydrogels. According to our recent findings, targeted drug delivery at a certain spot occurs due to external stimulation such as temperature, pH, glucose, or light. As an adjustable biomedical device, the hydrogel has tremendous potential for nanotech applications in involved health areas such as pharmaceutical and biomedical engineering. An overview of hydrogel characteristics and functionalities is provided in this review. We focus on discussing the various kinds of hydrogel-based systems on their potential for effectively delivering drugs that are made of polysaccharides.

## 1. Introduction

Hydrogels are a form of highly hydrophilic biomaterials with three-dimensional architecture that can retain a significant amount of water and swell without disintegrating. Hydrogels can be either synthetic, natural, or hybrid forms. Natural polymer hydrogels are those derived from naturally sourced polymers, including polysaccharides, polynucleotides, and proteins. Neutral, cationic, and anionic categories describe the chemical properties of natural sources of polymers. These polymers are easily accessible, ubiquitous, affordable, non-toxic, renewable, and have other appealing biological features.

On the other hand, synthetic hydrogels such as chemically cross-linked polymers with comparatively high mechanical properties include polyethylene glycol (PEG), polycaprolactone [1], and polyvinyl alcohol (PVA), which can resist superior mechanical loads despite poor bioactivity and inadequate biocompatibility. Hydrogels are usually applied in areas such as sustained or targeted drug release [2,3], tissue defect repair [4,5], wound healing [6], antibacterial agents [7], cell engineering, etc. [8]. The ability to transfer a drug to its targeted site with the least or no toxicity is crucial for the triumph or failure of a therapeutic approach. While the drug’s therapeutic efficacy and toxicity are vital, it is also crucial to choose a proper DDS to keep the drug along the route of administration and release it at the desired target site. Polysaccharides are mostly carbohydrates made up of multiple monosaccharide units bound together by glycoside linkage. They are a type of biological polymer that is bioactive, non-toxic, water-soluble, and biodegradable. These can be simply chemically and biochemically altered to increase bio adhesion with biomolecules, stability, and drug bioavailability [9]. There are low-cost methods for isolating polysaccharides such as algae (e.g., alginate), vegetation (starch), animals (e.g., chitosan), and microorganisms (e.g., Xanthan gum). These polymers have diverse chemo physical and biological properties that contribute to their excellent biocompatibility and bioactivity while being resistant to enzymatic degradation.

Furthermore, the presence and appearance of readily available functional groups such as carboxyl, hydroxyl, and amino groups enable drug conjugation and a range of chemical changes that are appropriate for the purpose [10]. Since our main aim is to comprehensively assess polysaccharide-based hydrogels for application in the DDS, we consider hydrogels as they are hydrophilic, three-dimensional (3D) systems that can incorporate massive amounts of either water or fluids, which make them ideal candidates for being used as biosensors as well as cell carriers in tissue engineering. Regarding the drug delivery approach, the porous structure of hydrogels can form a matrix for drug loading while protecting drugs from the harsh environment. Additionally, the permeability of bond strength in the hydrogel can change its properties. The releasing rate is another key parameter for drug carriers that is mainly determined by the molecule’s diffusion coefficient through the gel network and can also be modified to meet the required needs. Hydrogels’ chemical and physical structure can be designed to provide biocompatibility and biodegradability. All these qualities make hydrogels extremely promising for drug delivery [11]. In this article, we explain how polysaccharides have been employed in drug development, the properties of hydrogel design technologies, the formulation of polysaccharide-based hydrogels in the DDS, and hydrogel technical features. In addition, we add visions for the future and possible limitations of applying them in clinical use.

## 2. Materials and Methods

Classification of papers from accredited journals and electronic web services such as PubMed and Google Scholar was used to analyze what type of publications deal with our topic of “Polysaccharide-based Hydrogels with a Focus on the DDS”. After screening systematic and original English research articles, we selected 135 papers out of 220 potential studies to conduct this review. We began reviewing by briefly discussing the basic biochemical features of hydrogels and their production techniques. Following that, a detailed overview and in-depth analysis were conducted, emphasizing the impacts of various hydrogel properties on cellular functionalities and signaling activities. These functions or processes included basic properties of polysaccharide hydrogels, mechanism of formation, and possibilities for biomedical application. Initially, we conducted a non-systematic study of current concepts in polysaccharide hydrogels. References were identified through searches of the electronic databases in Scopus, PubMed and Google Scholar published mainly (not completely) between 2017–2022. We used polysaccharide-based hydrogels in DDSs (21 results) and polysaccharide-based hydrogels (52 results). Subsequently, this article was written based on an analysis of 135 research studies that were cited in potential updated papers of recent years. The cited references were thoroughly reviewed, and only the articles written in English were selected. Considering the originality and relevance to the scope of this review article, the final reference list was created. The keywords were initially filtered thus: “drug delivery”, “bio-based hydrogel”, “polysaccharide-based hydrogel”, “hydrogel”, “polysaccharide-based”, and “biomedical”, and then the last three mentioned keywords were excluded. The reason for writing this review was our experience in Professor Naimi-Jamal’s group with publishing on hydrogel-based DDSs and the invitation received from *Polymers* journal.

## 3. Technical Features of Polysaccharide-Based Hydrogels in Drug Delivery

**Figure 1:**
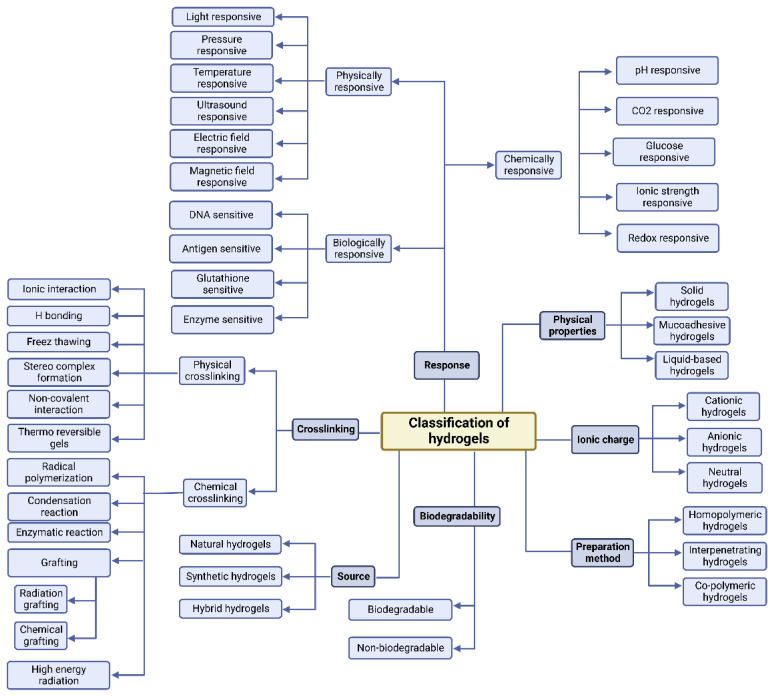
Classification of hydrogels according to their various characteristics.

**Figure 2:**
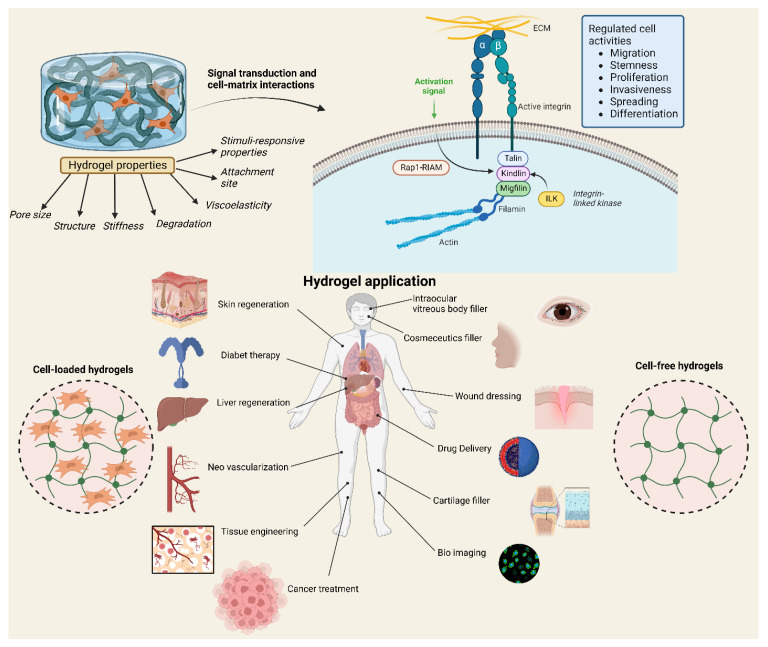
Hydrogel biomedical applications, properties, and interaction with cells.

Due to various bio-mimicking properties, hydrogels have been used in various biomedical fields. As illustrated in Figure 1, they have many significant properties and classes. In biomedicine, they are applicable in various areas such as disease model formation, cell culture, maps for tissue engineering, cancer treatment, carriers for bioactive agents, bioimaging, biosensors, and wearable technology application, and the most important aspect might be our aim to study hydrogels as a potent DDS. Hydrogels vary from other types of biomaterials in that they have a high water content, have a controlled swelling tendency, are simple to handle, and are relatively biocompatible, all of which make them desirable for biomedical applications. Due to their chemical structure and cross-linked network, they may react to a variety of stimuli that allows them to address the needs of a wide variety of biomedical applications as illustrated in Figure 2 [12]. According to the kind of polymer utilized, these stimuli may include heat, pH, light, and chemicals. Hydrogenated synthetic or natural polymers are considered the basic materials for medical applications. For some application areas where the hydrogel may be in direct contact with blood (synthetic or natural), it must also be blood friendly and biodegradable [13].

Once polymers are considered carbohydrate substances, they can extensively generate physical and chemical hydrogels owing to their availability, adaptive functional groups, and to some extent their biocompatibility [14]. Chemical cross-linking and physical cross-linking are the two ways to form hydrogels. Hydrogels can be personalized to a specific usage by selecting the type of monomer, polymer, and hydrogel formation techniques [15]. Figure 1 illustrates hydrogel classification based on various characteristics.

Polysaccharide-based hydrogels have sparked much interest in pharmaceutical and biomedical areas because they have many advantages, such as being biodegradable, biocompatible, and non-toxic in situ. Some other distinguishing characteristics of hydrogels are swelling behavior, elasticity, porosity, permeation, mechanical properties of their nanohydrogel form, biocompatibility, bioactivity, inhomogeneity, and the highest absorption under load [16]. As Pushpamalar et al. mentioned in their study, mechanical properties and toxicity tests must be assessed before they can be employed in the industry [17]. Several features can prioritize hydrogel application in the field, including the lowest soluble content and residual monomer and being cost-efficient as the sources of many hydrogels are not only natural but also available. Rostami et al. revealed that hydrogels have the highest durability and remain neutral after swelling and during storage; have the highest biodegradability without the formation of toxic species following degradation; are pH-independent for absorbing water; and are colorless, odorless, and absolutely non-toxic. Hydrogels also have light stability and re-wetting capability [18]. Meanwhile, hydrogels’ level of porosity has its roots in the system surrounding hydrogels, which is not simple to investigate. To clarify, ionic solutes can impact the porosity of hydrogels that uncharged solutes may have [19]. The following are some of the properties of hydrogels that are technically significant and should be considered in more detail.

### 3.1. Biochemical Characteristics of Polysaccharide-Based Hydrogels

Based on their chemical structure, hydrogels are classified into several classes: Polysaccharides (e.g., cellulose, starch, gums), biological polymers (DNA), polyamides (collagen), polyphenols (lignin), organic/inorganic polyesters, and polyanhydrides [13]. Essential aspects of the hydrogels include strength, stiffness, relative biocompatibility, biodegradability, ability to absorb water (swell), and stimuli responsiveness. These qualities are critical for electro–biochemical applications. As Varghese et al. revealed, both synthetic hydrogel and biological soft tissue are “soft and wet” materials since they are both spongy and wet. However, biological tissue, such as muscle, displays several types of functionalities, and hydrogels typically perform poorly [20]. This is partly because biological tissue has a complicated structure, whereas most hydrogels are amorphous.

Furthermore, the cytotoxicity of biocompatible material is crucial for its usage in biomedical utilization. Polysaccharide-based hydrogels, however, ought to be biodegradable and nontoxic. Biocompatibility is the capacity of a material to engage effectively with the host tissues and react accordingly in a particular setting. Bashir et al. represent that biosafety and bio-functionality are the major fundamental elements of biocompatibility. If the hydrogels do not comply with these requirements, they can become damaged. Toxic chemicals used to prepare hydrogel formulations frequently generate in vivo biocompatibility issues [10]. In addition, polysaccharides have been considered safe for food applications since they are non-toxic. In addition, biodegradable hydrogels are an absolute necessity in the biomedical industry. Likewise, polysaccharide-based hydrogels are considered biodegradable if organisms can break them down into inactive byproducts. The moieties are determined by the systems and the technique of synthesis. Degradation processes include hydrolysis and solubilization of biological entities of hydrogels to yield end products. Based on Ahmad et al.’s results, bio-absorption and bio-erosion may lead the hydrogels being disintegrated and easily removed from the body [21].

### 3.2. Chemically and Physical Crosslinked Polysaccharide-Based Hydrogels

Chemical cross-linkable hydrogels are a form of hydrogel that may be covalently bonded from a liquid to a solid. To produce hydrogels chemically, this approach employs many reactions, including optical polymerization, enzyme reactions, and click reactions. Because of their high mechanical strength, chemical cross-linked hydrogels have been investigated and employed in various areas such as pharmaceuticals, agriculture, food processing, and cosmetology [22]. Physical hydrogels are formed by interacting with molecular entanglements and/or additional forces such as ionic, H-bonding, or hydrophobic interactions. Since these connections are weak, physical hydrogels are classified as reversible gels. These are made without applying cross-linking reagent chemical changes. Physically cross-linked hydrogels are more susceptible to degradation. Based on the work of Parhi et al., unlike chemical crosslinked hydrogels, physical ones are homogeneous. These gels are very promising for introducing bioactive compounds [23].

### 3.3. Swelling Properties of Polysaccharide-Based Hydrogels

Polysaccharide-based hydrogels can potentially absorb liquids owing to the cross-linked polymeric materials in their structures. This capacity, based on ionic groups in the body—the larger number of ionic groups can lead to a higher capacity of holding water—plays a critical role in transferring nutrients and cellular products throughout the hydrogel and makes releasing drugs from hydrogels more efficient [24]. In addition, Suflet et al. (2021) showed that covalent association with physical cross-linking techniques could form hydrogels with the advantages of fast-swelling and low-elastic modulus [25]. Moreover, the equilibrium and swelling kinetics can be affected by various variables, including cross-linking ratio, ionic interactions, synthesis process, and polymeric chemical bonding. To assess the swelling qualities of hydrogels, the swelling ratio, which is the weight-swelling ratio of swollen gel to dry gel, is used. It is essential to consider that cross-linking determines the swelling ratio of a hydrogel. Hamdy et al. revealed that strongly cross-linked polymers exhibit a lower swelling ratio and poorly cross-linked polymers have a higher swelling ratio. Additionally, the presence of hydrophobic and hydrophilic groups and the chemical structure of hydrogels determines their swelling behavior. Polysaccharide-based hydrogels with more hydrophilic groups swell more than hydrogels with more hydrophobic groups [26].

### 3.4. The Elasticity of Polysaccharide-Based Hydrogels

Elasticity is another main characteristic of hydrogels derived from not only cross-linking and charge densities of the polymeric network matrix but also the accumulation of a cross-linked polymer matrix that can happen to hydrogels when the synthetic procedure is performed. Hence, hydrogels can save their basic forms after stopping forces from making strain [27]. In this regard, Qian et al. designed a simple and environmentally friendly process for making hydrogels from polysaccharides that can serve as novel drug carriers. A reversible chemical link was formed between carboxyethyl-modified chitosan (CEC) and aldehyde-modified hyaluronic acid (A-HA) loaded with doxorubicin to create the hydrogels. This elastic and self-healing hydrogel is an intriguing contender as a drug delivery carrier [28].

### 3.5. Mechanical Properties of Polysaccharide-Based Hydrogels

This characteristic of hydrogels arises from the degree of cross-linking in their structure that causes stiff hydrogels if there are many incidences of cross-linking in the structure, while few instances of cross-linking can cause soft hydrogels. Therefore, they may play a key role in the mechanical properties of hydrogels making them capable of performing functional activities, including repairing ligaments and tendons, wound healing, tissue engineering, DDS, and being an appropriate option for replacing cartilage structures [29]. In this case, Singh et al. reported the synthesis of an *Acacia* gum polysaccharide-based hydrogel for wound dressings with high mechanical strength [30].

### 3.6. Biocompatibility and Bioactivity of Polysaccharide-Based Hydrogels

Biocompatibility and bioactivity arising from the attendance of freely accessible groups such as carboxyl (–COOH), amino (–NH_3_), and hydroxyl (–OH) leading to some functional chemical adjustments are the reasons that make it possible for hydrogels to be used in the biomedical area of studies. This means the suitable hydrogels should not only pass the biosafety test but also provide systematic feedback that is fitted on the host cells and enclosed tissues [31]. For example, to develop controlled drug delivery systems, Ali et al. created a hydrogel made of citric acid cross-linked polysaccharide from *Salvia Spinosa* L. that is pH-sensitive, biocompatible, and non-toxic [32].

### 3.7. Inhomogeneity of Polysaccharide-Based Hydrogels

Homogeneity of hydrogels can be defined as a sort of hydrogel with uniform distribution of cross-linking in their matrix, and inhomogeneity of hydrogels that do not follow this rule are noticed as spatial inhomogeneity that can have negative impacts on the efficacy of hydrogels’ functions. To be more specific, uneven arrangement of cross-linking can cause diminishment of the visual appearance and strength of the hydrogel [33]. Kopač et al. found that hydrogels always exhibit an inhomogeneous cross-link density distribution, another imperfection that isn’t accounted for in the Peppas-Merrill equation. Rheological measures can be used to characterize the cross-link concentration of hydrogels, while LF-NMR analysis can efficiently assess the gel inhomogeneity in drug delivery systems [34].

### 3.8. Absorption under Load (AUL)

The highest AUL is a factor showing how much moisture can be absorbed by a polymer under pressure. Based on work by Kim et al., the thickness of surface cross-linking can positively affect the factor of AUL. In addition, if the time of surface cross-linking increases, AUL will also be improved, which is an important factor in DDSs [35].

### 3.9. Molding Time

Hydrogels can be created quickly from physical cross-linking that rapid ionic gelation, for example, can make clearer. However, chemical cross-linking may lead to more stable and persistent hydrogels. Zakerikhoob et al. made hydrogels according to in situ alginates, which had the potential to soak up liquid and gel them much more quickly than other sorts of hydrogels. It is important to say that such characteristics of hydrogels allow them to be regarded as perfect-matched options for use in pharmaceutical science as well as health-related fields [36].

### 3.10. Self-Assembled Supramolecular Polysaccharide-Based Hydrogels

Inspired by nature, self-assembled hydrogels regulated by weak, intermolecular interactions have garnered much attention for generating systems with ordered structures and functionalities. These efforts have resulted in various self-assembled functional materials, including liquids, elastomers, gels, and hard materials. Most biopolymers, including collagen and nucleic acids, use molecular conformations to generate higher-order patterns and respond to small changes in environmental stimuli. Human-made macromolecules having similar effects have been the subject of extensive research because of the novel properties they bring to the field. One of the earliest examples of these structures includes polymer hydrogels, cross-linked networks of macromolecules that undergo reversible transitions in reaction to minor environmental changes. Thus, supramolecular hydrogels are a self-assembled network structure created by non-covalent bonds. Because of their ability to undergo sol–gel and/or gel–sol transitions in response to minor changes in their surroundings, these hydrogels are considered smart. Hoque et al. revealed that stimuli-responsive hydrogels represent fascinating substances with potential uses in biomedical engineering, DDSs to improve innate tissue regeneration, and medical diagnostics imaging [37].

### 3.11. pH Sensitivity of Polysaccharide-Based Hydrogels

A pH-sensitive hydrogel is a gel construction that responds to pH alteration. Hydrogels may often either expand or shrink in response to a shift in the chemically reactive environment. Hydrogels can be created utilizing in situ polymerization processes, making them ideal for implementation into microfluidic devices. These pH-sensitive hydrogels have applications in creating pH-sensitive control valves, systems that can release a substance when the pH is changed. pH-responsive hydrogels are a biomaterial with advantageous chemical and physical features at certain pH levels. Polymer chains are linked with acidic or basic groups. Hydrogels can release drugs in three distinct ways: through diffusion, swelling, and chemically triggered methods. Most people are comfortable with the diffusion-regulated approach, which bases its drug release model on Fick’s law of diffusing. When the drug molecules’ molecular dimensions are much smaller than the pore size of the permeable hydrogels, the hydrogels’ permeability is proportional to their diffusion coefficient. When the porous structure in the hydrogels and the size of the drugs are close, the cross-linked polymer chains inhibit drug molecule release [38].

Consequently, when the swelling rate is greater than the release rate of the drug, the swelling controls drug release [39]; this includes water molecule absorption followed by drug desorption. The sensitivity of dry (glassy) polymer hydrogels to modifying shape and volume during hydration regulates the drug release rate, which controls the hydrogel content and cross-linking density. Hydrogels are structures that allow water or other physiological fluids to permeate their interfaces thanks to free intermolecular linkages. The swelling results from the tension created by the circulating solvent, which causes the space between the polymer chains to increase (polymer chain relaxation). After the drug has been slowly released, the swelling will go away due to desorption [40].

One exampleis transdermal drug delivery. The outermost layer of skin, termed as stratum corneum, has many features such as cohesion, intercellular lipids, permeability, etc. It is affected by many factors such as pH of the skin. The normal pH of skin is in the index of 5.0–6.0, and this is why the stratum corneum is considered an acid mantle. The acid mantle changes due to many factors such as age, gender, sebaceous glands, apocrine glands, eccrine glands, and epidermal cells. These factors lead to various disorders such as acne or inflammation. High skin pH causes micellization (>6.0) while a pH under 4.5 results in structural disorders. Patch dermal therapy is extremely crucial, especically to prevent side effects when longer administration is necessary. Hence, Kwon et al. prepared pH-sensitive hydroxyethyl cellulose/hyaluronic acid (HECHA) composite hydrogels cross-linked with divinyl sulfone to control drug release of isoliquiritigenin (ILTG) to treat propionibacterium acnes [40].

### 3.12. Polysaccharide-Based Hydrogels with Temperature-Sensitivity Feature

It has been discovered that the tumor, ischemia, and wound healing sites are acidic. Therefore, researchers have been motivated to create medication delivery methods that may specifically target areas of local acidosis through dual pH and temperature-sensitive hydrogels. The temperature-sensitivity in thermally sensitive hydrogels is mediated by the delicate balance of hydrophobic and hydrophilic components of the polymer monomer, which has both hydrophobic and hydrophilic aspects in its framework [41]. The dissolution of the cross-linked system and the sol–gel phase separation are modified as a function of temperature due to changes in the interactions of the hydrophilic and hydrophobic segments of the polymer with water molecules. The gel phase is stable and does not migrate compared to the moving sol phase. The macroscopic dissolved phase of a cross-linking network in an aqueous solution is identified by shifting the balance of hydrophilicity and hydrophobicity. Mechanisms on a micro level related to thermo-sensitive subunits can be employed to obtain the gelation capability of thermo-sensitive hydrogels at either the lower critical solution temperature or the upper critical solution temperature (UCST); hydrogels separate from the solution and solid. The polymer is soluble in the presence of a lower critical solution temperature (LCST), but it begins to shrink, becoming hydrophobic and insoluble, in the presence of an LCST, leading to gel formation. Instead, the UCST can be found in the hydrogel formed when the polymer solution is cooled. Specifically, the polymer in solution undergoes a phase shift, changing from a soluble (random coil) to an insoluble state (collapse or micelle form) as it approaches the critical temperature. The ratio of hydrophilic to hydrophobic groups determines the LCST. Hydrogels that release their bioactive ingredients constantly based on temperature have seen significant development [42]. Thermo-sensitive gels offer many benefits as a delivery mechanism. Despite typical hydrogels that must be surgically placed, the temperature-sensitive properties of the hydrogel enable delivery, preventing first-pass metabolism. The heat-responsive gel is preferable for injectable applications because it does not require any denaturing cross-linking agent; additionally, the temperature-induced sol–gel transition is entirely safe when occurring inside the body. Encapsulation in a flowing form provides homogeneous dispersion of therapeutic drugs in hydrogels. In contrast, quick sol-to-gel transition at body temperature avoids early burst release of therapeutics, allowing for controlled-release behavior. Moreover, the flowable administration gives the hydrogel form stability [41].

### 3.13. Affinity of Polysaccharide-Based Hydrogels

The functionalization of hydrogels with ligands results in affinity hydrogels (heparin, peptides, and aptamers are a few examples). Because of the strong protein–ligand binding, affinity hydrogels can retain protein permanently. They primarily control protein or drug molecules released through a diffusion-coupled binding reaction. Using particular activating molecules, affinity hydrogels can be designed to gain biomimetic intellect for on-demand protein release [43]. Rial-Hermida et al. reported that the mechanism of affinity-based delivery exploits the interactions between the biotherapeutic drug and the delivery device. These interactions can be advantageous bilaterally, in both incorporation and release of active drugs. In these instances, the release can be controlled by the intensity of the affinity contacts, the concentration of the binding ligand, the characteristic of dissociation of the synthesized complexes, and the size and shape of the hydrogel [44].

## 4. Hydrogel-Based Drug Delivery Systems

Research into smart hydrogels, which can monitor their environments and modify their behavior, has developed in the biomedical engineering field in recent years. Smart hydrogels are a promising material for DDSs because of their high stability, physicochemical characteristics, and biocompatibility. Hydrophilicity, swelling capacity, physical properties, and molecule permeability can all be modified in smart hydrogels in response to environmental factors such as pH, electrical and magnetic fields, temperature, light, and the levels of biological molecules, allowing for a more gradual and predictable release of the drugs payload. It has been shown that hydrogel-based systems are an efficient method for achieving regulated drug delivery. Hydrogels, which can be formed from cross-linked polymers, are commonly used as carriers in controlled-release systems because of their unusual release mechanisms, including diffusion and swelling. The most common and straightforward hydrogel-based dosage forms are tablets intended for oral administration. For their manufacture, various excipients, such as a swellable polymer and a drug, must be combined and compressed in the right proportions.

There has been a rise in the development of smart biomaterials in recent years in response to the increasing interest in personalized medicine plans. Active biomaterials include hydrogels that alter their properties in response to external stimuli such as pH, temperature, electrical and magnetic fields, light, and biomolecule concentration. There is evidence that the terms “stimuli-responsive hydrogel” and “smart hydrogel” were initially published in the scientific literature in 1990 and 1991, respectively [45]. In response to a modest external trigger, smart hydrogels demonstrate sudden changes in physical characteristics and macroscopic modifications. What makes these hydrogels special is the nonlinear feedback they produce. They can respond to triggers by undergoing a phase-volume transition that is reversible, intensity-scalable, reproducible, and predictable. Furthermore, they can revert to their original shape once the trigger is withdrawn. Physical state, solvent interaction, solubility, conductivity, and hydrophilicity are only some properties that might shift during these phases [46].

In their research, Patel et al. used a new micellization approach to generate a biodegradable thermo-responsive hydrogel with enhanced stability. A triblock co-polymer was used with varying physical properties to obtain a critical solution temperature and critical gelling concentration that can construct a stable hydrogel network at body temperature. The results showed that the hydrogel was an excellent technique for maintaining drugs for disease treatment, as it prolonged the release of diclofenac sodium by about one hundred. The release is mediated mainly by diffusion via the hydrogel’s porous membranes [1].

The protein folding and unfolding method was used by Qingyuan et al. to build a layered protein-based shape memory/morphing hydrogel. They constructed the protein-bilayer structure using two simultaneous modular elastomeric proteins (GB1)8 and (FL)8. The denaturant-dependent swelling profiles and Young’s moduli of the two protein layers are different. Because of the swelling variations caused by the unfolding and refolding of proteins, the bilayer hydrogels could be bent in either direction with a great degree of control over the amount of denaturant and the shape of the layers. Utilizing these controllable and reversible bending behaviors as a foundation, the scientists folded patterned hydrogels from one to two and three dimensions by using the protein-bilayer structure as a hinge [47].

Using free radical polymerization in the presence of Poly (NIPA-co-VSA) nano gels, Kaan Emre et al. presented a pH and temperature-responsive poly/Alginate interpenetrating polymer network hydrogel. Physical cross-linking of the hydrogels with the Ca2+ ions was achieved by using Na-Alginate, a natural polymer. Mechanical studies revealed a wide range in fracture strengths, from 137 to 830 kPa. Using the solution impregnation technique, the doxorubicin (DOX) loading capacities of the hydrogels were calculated to be between 86 and 161 mg DOX/g polymer. Their DOX release characteristics have been studied in relation to pH, temperature, and the degree of physical cross-linking of hydrogel [48].

Therefore, hydrogels’ functionality can be improved, and their potential applications in biomedical engineering can be expanded by including stimulus-responsive effects. A developing family of materials called “smart hydrogels” can respond to environmental cues, including pH, temperature, electrical and magnetic fields, light, and biomolecule concentration to release the drug cargo precisely where and when it is needed. Considering the research conducted over the past five years, scientists want to focus on enhancing the qualities of existing smart hydrogels and adding new, cutting-edge capabilities to them soon. Synthesis of smart hydrogels that can integrate multiple therapies and respond to complicated, multiple stimuli will be a focus of future studies. Even if there are certain difficulties in this area, their future is promising. Since their beginning, numerous smart hydrogels have been studied, developed, and presented; yet, there is insufficient data to support the commercialization of smart hydrogels as DDS. However, a select group has made it into clinical use, with UroGen Pharma’s Jelmyto^®^ (UGN-101) having been introduced and receiving FDA approval (2020). Despite recent developments in the pharmaceutical sector, there are currently no established regulatory norms and standards for the therapeutic use of smart drug-loaded hydrogels. It is also crucial to simulate the release profiles prior to commercialization, which will allow for significant advancements around in vivo release. The potential for smart hydrogels to revolutionize 21st-century medicine is evident, and the field is still in its infancy. It is expected that gene-loaded hydrogels with built-in sensors will be the primary focus of future generations of smart hydrogels for treating genetic disorders. Pathogen-responsive hydrogels as a potential treatment for infections at a local level are another subject for future study. The European Research Council (ERC) has recently sponsored a project called “Gels4Bac,” which will investigate the selective and local release of antimicrobial vesicles in response to particular pathogen triggers [45].

### 4.1. Polysaccharide-Based Hydrogels

Polysaccharides are unique natural polymers with a wide range of structural traits. Because of their superior biological characteristics, polysaccharides can be utilized as regenerative biomaterials. They are composed of long-chain carbohydrates of repeating monomeric units linked together through glycosidic bonds. These are potential biomaterials with unique physiological functionalities and biological activities that can be used in various fields. Polysaccharides naturally found in the environment, such as cellulose, starch, dextran, pullulan, and pectin, are being extensively researched for medicinal, pharmaceutical, and biomedical engineering uses. During the last few years, there has been a lot of attention focused on creating and improving polysaccharide hydrogels for biological purposes. Drug-loaded hydrogels can maintain serum levels and can be administered through injection, orally, or intramuscularly. Despite the importance of synthetic biocompatible and biodegradable polymer hydrogels for biological applications, polysaccharides continue to be smart and appealing due to their wide range of applications, non-toxicity, good biocompatibility and biodegradability, cost-effectiveness, simplicity of modification and preparation, high efficiency, and renewable physio-chemical properties [10].

### 4.2. Hydrogel-Based Controlled and Extended-Release Systems

Clinical applications have shown that hydrogel-based controlled-release systems can take advantage of therapeutically beneficial drug delivery outcomes. Hydrogels allow for the precise regulation of the release of a wide range of therapeutic agents, from small-molecule drugs to macromolecular pharmaceuticals to cells. Due to their malleable physical qualities, variable degradability, and capacity to preserve labile drugs from degrading, hydrogels provide a substrate upon which diverse physio-chemical interactions with the encapsulated drugs regulate their release [49].

Cross-linking linear polymers or polymerizing monofunctional monomers and cross-linking with bifunctional monomers simultaneously are necessary to prepare hydrogel-based therapeutic products. The mechanical strength of weakly cross-linked hydrogels can be improved in sufficient ways. Hydrogels can be synthesized using polymers derived from natural, synthetic, or semi-synthetic sources. Polymers with functional groups including hydroxyl, amine, amide, ether, carboxylate, and sulfonate on their side chains are frequently employed. A full list of monomers and cross-linkers is available in the literature [50].

Controlled-release polymeric systems are often categorized as either “matrix” or “reservoir”. The simplicity, low cost, and superior performance of matrix systems have made them the de facto standard in their field. However, such systems often use Higuchi’s model, in which drug release is proportional to half-life. This results in varying release rates, which are lower initially and fall off more sharply later on. The nearly consistent release rates are the primary benefit of hydrogels for controlled drug administration [51].

Most hydrogels are glassy when they are dehydrated, and drug release typically occurs through simultaneous water absorption and desorption via a swelling-controlled mechanism. The polymer’s ability to resist swelling and deformation mediates drug administration at a rate controlled by the drug’s concentration. When a glassy hydrogel is exposed to water or another thermodynamically suitable media, the solvent can penetrate the interstitial spaces between the macromolecular chains. If enough water is included into the matrix, the glass transition temperature of the polymer will decrease to the value determined experimentally. In a glassy polymer, the presence of solvent leads to the production of stresses that are accommodated by a rise in the radius of gyration and end-to-end distance of polymer molecules; this is macroscopically manifested as swelling. There is a clearly defined velocity front as solvent molecules travel into the dry (glassy) polymer matrix, while the thickness of the swelling (rubbery) region grows with time in the opposite direction. This type of expansion and spreading does not typically occur by a Fickian diffusion mechanism [52].

Polymers containing ionic pendant groups form the backbone of hydrogels that change their pH in response to their environment. Poly (methacrylic acid) (PMAA), poly(diethyl aminoethyl methacrylate) (PDEAEMA), poly (acrylic acid), poly(acrylamide), and poly(dimethyl aminoethyl methacrylate) (PDMAEMA) have all been extensively researched for their pH-responsive behavior [48]. This is caused by the pendant groups ionizing and developing fixed charges on the polymer network. The drug release can be modulated by modulating the swelling or deswelling of the hydrogel in aqueous environments of varying pH and ionic strength [53].

Fang et al. developed pH-responsive and magnetic carboxymethyl starch/alginate hydrogel beads (CMCS-SA) containing the MgFe_2_O_4_ nanoparticles to release the anticancer drug DOX in GI-fluid-like conditions. In vitro release behaviors further confirmed the beads’ high stability in the stomach-like fluid. On the other hand, data from simulated intestinal fluids demonstrated persistent DOX release due to the pH-sensitive swelling features of the fluids. Notably, an external magnetic field (EMF) applied to the beads may hasten the release of the medicine. Diffusion, swelling, and erosion were the primary mechanisms responsible for the in vitro release of drugs from gel beads. Drug-loaded hydrogel beads were very cytotoxic to HCT116 colon cancer cell lines but had no effect on normal 3T3 cells in a cytotoxicity test. As a result, the produced gel beads may be qualified as dosing platforms for anticancer medicines [54].

Similarly, Fernanda et al. produced a biodegradable and multicompartmental hydrogel for the controlled-release of hydrophilic (DOX) and hydrophobic (niclosamide) pharmaceuticals by combining N-isopropyl acrylamide, cellulose, citric acid, and ceric ammonium nitrate. Research shows that cellulose slows the release of drugs, with only 4% of DOX and 30% of niclosamide released after 1 week. Despite the minimal release level, cell death occurred in both cell lines. In addition, this hydrogel showed the ideal characteristics of injectability, in situ prevalence, and safety in vivo. In summary, the hydrogel’s qualities and its natural and environmentally friendly composition produce a reliable and effective platform for the regional treatment of tumors that cannot be surgically excised or require adjuvant therapy before surgical removal [55].

Suhail et al. also used free radical polymerization to make hydrogels out of glutamic acid and polyvinyl alcohol. The hydrogels were pH-responsive, with the hydrogel size increasing and drug release rates changing when the medium pH was increased from 1.2 to 4.6 and 7.4. Furthermore, drug loading and porosity percentage were measured for the manufactured hydrogels. When the percentage of glutamic acid and acrylic acid in the matrix was increased, porosity and drug loading were both shown to rise, but the opposite was observed when polyvinyl alcohol was increased. Sol–gel analysis showed that as glutamic acid, polyvinyl alcohol, and acrylic acid concentrations in the hydrogels were raised, the degree to which they cross-linked increased while the degree to which they un-cross-linked decreased. Hydrogel degradation slowed with increasing glutamic acid, polyvinyl alcohol, and acrylic acid concentrations, suggesting that hydrogel networks formed when high hydrogel contents were stable. Similar to in vitro studies, in vivo studies have shown that the created hydrogels can release their drugs slowly over time, making them a viable controlled DDS [56].

Novel approaches to synthesizing bioactive compounds for topical administration are of ongoing interest in the pharmaceutical industry, as Yohana et al. have demonstrated. Their work contributes to a better understanding of bigels, which are matrix-in-matrix systems formed when a hydrogel and an organogel are combined. The potential precursors tested were collagen, hypromellose, alginate, gelatin, sesame oil, isopropyl myristate, and medium-chain triglycerides. However, the consistency and uniformity of the bigels are dependent on the composition of the initial materials and the processing circumstances. Contrarily, as shown by the diclofenac dissociated and non-dissociated tests, bigels have a distinct structure from the beginning gels, emulgels, and other similar systems, which governs their rheological and textural characteristics and modulates the drug distribution. This semisolid solution can be useful in creating a wide variety of pharmaceutical products for which one of the main points is the controlled-release of active pharmaceutical ingredients [57].

Wenxiu et al. developed Pickering emulsion hydrogels (PEHs) as a pH-responsive, controlled-release delivery technology to overcome the shortcomings of Pickering emulsions under certain severe processing or gastrointestinal circumstances. The PEHs were developed using a matrix of alginate and gellan gum (GG) with carboxymethyl chitosan (CMCS) at varying concentrations. The PEHs with 0.8% GG [58] had better texture profile analysis features and Young’s modulus. In vitro, the emulsions were not released at pH 2.0 due to the presence of the PEHs; they were completely released at pH 7.4 due to the action of CMCS and GG concentration. This research paves the way for the regulated release of hydrophobic actives in biomedical settings via Pickering emulsions that exhibit high stability and a pH-dependent release profile [59].

### 4.3. Nano-Systems for Polysacharid-Based Hydrogels

According to their particle size, polymeric hydrogels are categorized as macro-, micro-, or nanogels. The size of microgels’ cross-linked structure is on the big side, measuring in millimeters or centimeters. Microgels are a class of cross-linked gels in which the particle size is between 0.1 μm and 100 μm. Any gel with a particle size of less than 100 nm is considered a nanogel. Notably, quasi-nanogels were defined as gels with particle sizes just slightly larger than 100 nm. Microgels are a type of network polymer that can be either submicron or micron in size [60]. Particle form and size can be precisely controlled in cross-linked microgels, perhaps because of their unique, widespread swelling pattern. There are two methods to obtain microgels. For example, existing polymer molecules can be assembled into larger structures in aqueous solutions. Two distinct polymerization processes contribute to the formation of particles: precipitation polymerization and inverse emulsion polymerization [61].

**Figure 3:**
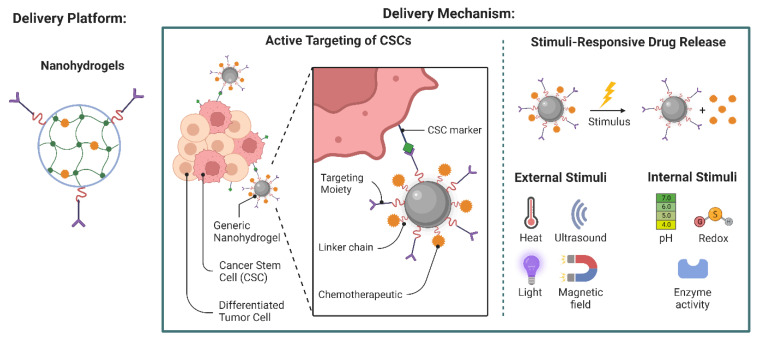
Nanoparticles directly deliver drugs to cancer stem cells (CSCs). These cells, known as CSCs, can self-renew and differentiate into a wide variety of tumor cell types.

Nanogel particles, which are biocompatible and biodegradable, are easily able to traverse the blood–brain barrier because of their swelling and deswelling capabilities and small size (Figure 3). Water-based nanogels are often made from natural polysaccharides (dextran, pullulan) or a polysaccharide containing cholesterol. Typically, the size of these hydrogels is between 20 and 30 nm. As a result of their small size, nanogels are utilized for cell targeting, and the entrapped drug is released due to swelling produced by changes in pH in the surrounding environment. Compared to microgels, nanogels have a far faster response time to alterations in external stimuli, probably due to their diminutive stature and brief resting period [62].

It is challenging to obtain hydrogels in a precise shape at the nanoscale. Electrostatic spinning is one approach for producing hydrogels nanofibers. Microcapsules and microspheres, ranging in size from 1 to 1000 μm, are another hydrogel delivery form. Substances housed within microcapsules and microspheres benefit from this increased level of security. Micelles can be formed in water by aggregating amphiphilically linked blocks or end-modified polymers, as with hydrogels. Since reversible hydrogels can form micelles above a suitable concentration, known as the micelle gel concentration, distinguishing micelles from reversible hydrogels is often challenging. Drug molecules are absorbed on the surface or enclosed inside nano-capsules or biodegradable nanospheres [63]. Controlled delivery of hydrophobic drugs via chondroitin sulfate hydrogels has been a focus of research and development in recent years due to the availability of biopolymer-based injectable hydrogels [64].

Another class of hydrogels is the in situ-gelling kind, which undergoes a sol–gel transition in vivo. This hydrogel gang always changes its form to fit the available area. Shear-thinning hydrogels are one example of this type of hydrogel that softens when subjected to shear stress; they can restore their original stiffness when injected outside the body thanks to reversible physical cross-links. Though there are benefits to using nano- and microgels, it is important to remember that microporous hydrogels may also be an option. After being injected into a human, microporous hydrogels can undergo a mechanical collapse of up to 90% and then rebound practically instantly. Because of their microporous structure, the creation of precisely shaped drug delivery vehicles is feasible [65]. These systems are of special relevance from a drug delivery standpoint since their synergistic effects are exhibited as nanocomposites, while the drug delivery restrictions of microgels and hydrogels are mitigated. As a result, utilizing hydrogels as carriers for active compounds has benefits. To begin, the release time of hydrogels can be altered to be either slow or quick. This function can significantly aid patients’ adherence to their treatment plans. Moreover, customized hydrogel materials allow for on-demand control of drug release. As a result, the entire hydrogel network structure can react to changes in pH or the presence of specific ions by adding enzymes or ionizable groups [66].

### 4.4. Dual-DDS Based on Hydrogel/Micelle Composites

Polymeric micelle, a DDS self-assembled from amphiphilic block or graft co-polymers, is another notable DDS. These polymeric micelles have been found to have unique stability in aqueous environments. Micelles’ core-shell structure enhances the solubility of hydrophobic medicines and prevents the integrated drug from degrading too quickly, as has been shown in previous studies. These amphiphilic co-polymers can be employed as an environmentally controlled drug release mechanism when functional groups sensitive to environmental conditions are included in the molecule. For instance, Ko et al. encapsulated DOX in methyl ether poly (ethylene glycol)-poly (-amino ester) block co-polymer micelles. Indicative of pH-dependent micellization–demicellization activity, these micelles exhibit rapid release at pH 6.4 and a slow release at pH 7.4 [67].

Combining drugs with various therapeutic effects has recently been shown to be an efficient method for treating diseases and regenerating damaged tissues. In combination therapy, controlling the release behavior of each drug is a significant problem. Different drugs should be administered at their appropriate doses and times in the treatment to maximize their effects. However, standard drug delivery methods fall short of what this kind of treatment requires. Therefore, creating dual DDSs that allow for tunable drug release is desirable. There have been few published studies on this DDS. For instance, Lee et al. created a straightforward dual-drug-loaded hydroxypropyl methylcellulose (HPMC) matrix tablet containing drugs in both the tablet core and the coated layer. The generated biphasic release profile HPMC matrix tablet can be used to provide medicines whose physiologic effects are time-dependent [68].

Hydrogel/micelle hybrid systems were proposed as a delivery mechanism for a dual-drug release vehicle in a study by Wei et al. Poly (vinyl alcohol) (PVA) or a chitosan (CS)/PVA mixture is used to make the hydrogel. PVA hydrogel is used due to its favorable physical-mechanical qualities, while CS hydrogel is employed because of its sensitivity to changes in pH [69]. For drug distribution, both hydrogels show high biocompatibility. Poly -b-poly -b-poly (PLGA-b-PPO-b-PLGA, abbreviated as GPG) is used to make the pH- and temperature-sensitive micelle. Examples of model drugs include the fat-soluble DOX and the water-soluble aspirin. Hydrogels contain Asp that has been directly disseminated, while GPG micelles contain DOX that has been encapsulated. The effects of pH and temperature on the drug release behaviors of GPG micelle, PVA/micelle DDS, and CS/PVA/micelle DDS were investigated. To learn how drugs are released into the body, a power law equation was applied to drug release profiles [70].

Cong et al. presented a new chitosan-based cross-linked unimolecular micelle in their research. The pH-sensitive hydrogel/micelle composites were prepared by loading emodin (EMO)-encapsulated micelles into a sodium alginate hydrogel matrix. Combining Box–Behnken experimentation with response-surface methods led to the development of an optimal micelle formulation with 8.06% CaCl_2_, 1.71% chitosan, and 26.52% -GP. The micelles’ diameter increased from 80 nm in aqueous solution to 100–200 nm in the hydrogel due to the formation of polyelectrolyte complexes, as revealed by morphological examination. The physical properties of simulated digestive fluids were studied, and it was found that the ratio of hydrogel to micelle significantly affected swelling, degradation, and in vitro drug release behaviors. The sustained-release profile of the 1:1 hydrogel/micelle mixture was observed, while the colon-specific profile of the 3:1 mixture was observed. Drug release from these two formulations was found to follow a complex process in which multiple processes were involved or occurred simultaneously, as evidenced by their matching release mechanisms. The study’s results show that pH-sensitive hydrogel/micelle composites made from biocompatible materials can be an effective sustained-release or site-specific drug delivery strategy for unstable or hydrophobic medicines [71].

Camptothecin (CPT) and granulocyte colony-stimulating factors were co-encapsulated and released slowly over time in another study by Ma et al. using a novel supramolecular hydrogel/micelle composite. Micelles with CPT-loaded hydrophobic cores and G-CSF-complexed hydrophilic shells were self-assembled using heparin-conjugated Pluronic F-127 produced by a click reaction. An injectable supramolecular hydrogel/micelle composite was produced under mild circumstances by combining α-cyclodextrin with CPT-loaded Hep-F-127 micelle/G-CSF complexes in an aqueous solution. Co-encapsulated CPT and G-CSF were able to retain biological activity and display a longer release compared to CPT-loaded Hep-F-127 micelles/G-CSF complexes. In addition, the amount of α-CD used allowed for fine-tuning of gelation characteristics and drug release profiles [72].

Anidruhan et al. presented a hydrogel/micelle composite as a dual-drug release vehicle in a different investigation. This hydrogel is made by mixing poly(Ethylene glycol) (PEG) and poly (vinyl alcohol) (PVA). Many biomedical industry researchers are looking at polymeric micelles as a potential solution for the stability and distribution of water-insoluble drugs. Micelles made from the co-polymer of oleic acid and g-chitosan were designed and synthesized with the help of this characteristic. The pain reliever Tramadol and the antibiotic Cefixime trihydrate were employed as prototype drugs. The drug release characteristics of the micelle and PEG-PVA/micelle DDS were analyzed as a function of pH and temperature. A power law equation was used to examine the release profiles and determine how the drugs were released. The research was conducted on the drug delivery vehicle, and properties such as swelling, ionic strength effect, antioxidant activity, microbiological stability, and in vitro drug release were identified. The two medicines were released in a much greater quantity in the basic medium than in the acidic medium [73]. To increase 5-FU’s efficiency against skin cancer and decrease its systemic side effects, Pourmanouchehri et al. devised a pH-responsive micellar hydrogel system based on deoxycholic acid micelle (DCA Mic) and carboxymethyl chitosan hydrogel. Results from experiments on drug release demonstrated that Hydrogel’s characteristics varied with pH. Melanoma cell proliferation was inhibited more effectively by the final formulation than by 5-FU. The 5-fU miconazole delivery platform shows promise for improved efficacy and reduced systemic toxicity in skin cancer treatment [74].

To achieve prolonged, stimuli-driven, and slow-release localized drug delivery, Chen et al. created cellulose-based biocompatible, tunable, and injectable hydrogels embedded with pH-responsive diblock co-polymer micelles. They made oxidized carboxymethyl cellulose (CMCCHO) with different degrees of oxidation in addition to hydrazide-modified carboxymethyl cellulose (CMC-NH_2_). In addition, atom-transfer radical polymerization was used to create pH-sensitive poly (ethylene oxide)-block-poly (2-(diisopropylamine) ethyl methacrylate) (PEO-b-PDPA) co-polymers as micelle cores to transport hydrophobic compounds (ATRP). An injectable hydrogel composite system was created by combining polymer suspensions of CMC-NH_2_ and CMCCHO, including PEO-b-PDPA co-polymer micelles in a Schiff base reaction. The release test using Nile Red dye and DOX demonstrate a pH-triggered, extended, and slow-release profile from this newly manufactured, adjustable, cellulose-based double barrier system. The hydrogel system also saw similar storage moduli and controllable degradation [75].

Polymeric micelle/nano hydrogel composite matrix is introduced as a novel multi-drug carrier by Anidrudhan et al. in another work. They found that combining these two drugs makes possible improved efficacy with reduced toxicity. Polymeric micelles, however, can include two drugs and release them concurrently without any premature leakage. Monomers with long alkyl chains, such as trimethylene tetraamine and oleic acid, were grafted onto the heparin (HEP) skeleton to boost the core and hydrophobicity. Folic acid was encapsulated in a hydroxyl appetite (HAP)-based hydrogel and then applied to polymeric micelle to facilitate drug targeting (PM). Similar investigations have shown that this coating could also be a potential barrier to sustaining the drug release. Studies of in vitro swelling and release were encouraging, with folic acid demonstrating burst release followed by sustained release of chemotherapeutic medicines. Therefore, the current material may serve as a highly effective, low-toxicity candidate for the treatment of colorectal cancer [76].

Lv et al. reported an artificial insulin administration system that achieved real-time glycemic control and reduced risk of hypoglycemia by simulating physiological basal and prandial insulin secretion. Micelles loaded with insulin were placed in a hydrogel matrix to create a glucose-responsive insulin delivery system based on phenylboronic acid and galactosyl [77]. The hydrogel and the micelles swell at the hyperglycemic state, emulating prandial insulin secretion by rapidly releasing insulin in response to elevated glucose levels. Only a subset of the micelles fully responded to glucose and continued to secrete insulin at a slower rate even after the glucose level had returned to normal. Hydrogels with higher cross-linking densities may mimic natural basal insulin secretion by releasing insulin slowly, as seen in the body. In a mouse model of type 1 diabetes, our hydrogel–micelle-composite insulin delivery device rapidly reduced blood glucose levels and kept them normal without hypoglycemia for around 24 h. Insulin delivery using a glucose-responsive hydrogel–micelle composite may be effective for treating diabetes [78].

## 5. Biomedical Applications of Polysaccharide-Based Hydrogels

Since hydrogels have hydrophilic groups in their structure, which can be linked with water molecules and hydrophobic groups, they can swell after absorbing water. As a result, Narmani et al. and Hyder et al. investigated the methods of preparation of hydrogels based on different materials to realize their importance for biological applications. Over the last years, polysaccharide-based polymeric biomaterials such as hydrogels have played an important role in designing and improving DDS. These benefits can come from their perfect properties: not only biocompatibility, bioavailability, and nontoxicity but also biodegradability and swelling, and it is easy to work with them as well. As a result, it is valuable to say that hydrogels can be regarded as proper carriers for drug delivery in oral, rectal, nasal, and vaginal routes [79].

Moreover, they have great potential to be used in DDS via skin for transdermal drug delivery applications. In addition, hydrogels may be able to sustain the release of drugs when they are utilized in tissue engineering and cancer therapy [80]. This shows that hydrogels can be considered a perfect-matched option for use in various fields such as anticancer drugs, contact lenses, regenerative medicine, tissue engineering, barrier material to regulate adhesions, food packaging, and controlled DDS that derives from the high water content and hydrophilic nature of hydrogels. In this next sections, the applications of polysaccharide-based hydrogels in biomedical fields are displayed precisely.

### 5.1. Topical Drug Administration

Hydrogels can protect patients from irritation coming from topical inflammation. This is because hydrogels have capability of maintaining high water content deriving from their large capacity, which can result in not only playing roles as moisturizers but also saving patients from scaling and dryness of the skin during treatments. According to Ravani et al., a clotrimazole hydrogel used for vaginitis via this delivery method has had a higher rate of absorption than conventional formulations [16].To clarify, a collagen hydrogel mask can be regarded as a perfect example of DDS by hydrogels that have a moisturizing effect to keep the skin as elastic and shiny as normal and healthy skin [81]. Moreover, Kumar et al. indicated that the Clobetasol propionate-based nano-sponge in combination with CAR-934 hydrogel can bring some benefits, including managing the release of CP (86% over 24 h) that allows this system to be used for topical administration (NS) [82]. Chen et al. used the IR780-LS in hydrogel to deliver d IR780 iodide as a tumor-targeting photosensitizer and IR792 perchlorate as a tumor non-target photosensitizer that, finally, brought some benefits including non-toxicity and high anti-cancer efficacy that stem from high anti-cancer concentration in target sites. Consequently, this method can be used to target tumors systematically via the topical route of administration [83]. In order to intervene in tendinopathy early on, Hsiao et al. used drug-loaded hyaluronic acid hydrogel as a prolonged-release strategy including dual effects. They demonstrated that tendinopathy can be cured using a combination of drugs that target multiple pathways in the disease’s aetiology and that are delivered in the form of a hydrogel [84]. Injectable hyaluronic acid hydrogels containing medication nanocrystals for the long-term therapy of inflammatory arthritis were also presented by Gao et al. This hydrogel may provide a response for intra-articular therapy of inflammatory arthritis in both in vivo and in vitro models [85].

### 5.2. Ocular Drug Delivery

During research on polysaccharide-based hydrogel, it has been proved that hydrogel can be applied for ocular DDS, which has benefits compared to other delivery systems, including raising the rate of drug absorption via the cornea, which leads to upgraded drug efficacy. Diclofenac can be considered as an ocular drug that has been delivered through hydrogels with the benefits mentioned before. Li et al. have gathered information about hydrogels that led to realizing that being biocompatible and having normal water content may facilitate the slow release of drugs much better compared to other sorts of materials in the ocular area. Diclofenac Sodium was one of the ingredients that was tested. As a result, hydrogels can be used in contact lenses, which are a perfect method for ocular drug delivery [86]. Using hyaluronic acid-PEG-based Diels–Alder in situ producing hydrogels, Ilochonwu et al. proved the efficacy of antibodies in the treatment of retinal disorders. In their study, the 4APM-HAFU hydrogel formulation (ratio 1:5) displayed prolonged release of bevacizumab > 400 days with a combined effect of diffusion, swelling, and degrading, indicating promising therapeutic antibody sustained-release potential [87].

Additionally, hydrogels have played important roles in clinical ophthalmology as drug eluting for soft contact lenses and intraocular lenses (IOLs). Hydrogels also can be used for the goal of controlled release via encapsulating ophthalmic drugs that can lead to the treatment of diseases such as glaucoma. For ocular delivery, hydrogels made of N, N-dimethyl acrylamide, poly-HEMA, and 2- (N-thyl per fluorooctane sulfonamide) ethyl acrylate have been effective in increasing the rate of absorption through the cornea. Lacrisert and vitrasert, created from hydroxypropyl cellulose, are examples that have shown benefits in treating dryness. To clarify, Cascone et al., 2019, named hydrogel cellulose as a beneficial choice for biomedical applications, including eye drops that stem from the characteristics of being hydrophilic and erodible enough [45]. In addition, they confessed that hydrogels have advantages that make them perfect options for ocular DDSs over other kinds of materials. Among these superior factors, high water content and no need for harsh situations such as high temperature to be produced are two factors that result in delivery of proteins or nucleic acids to the eyes.

### 5.3. Hydrogels in Colonic Drug Delivery

Polysaccharide-based hydrogel has some special properties that make hydrogels suitable for drug delivery to an organ such as the colon to treat diseases including colon cancer, ulcerative colitis, and Crohn’s disease. This is because in the colonic part of the human body, many polysaccharide enzymes make it possible for hydrogels to be used for colonic drug delivery and release their drugs in response to the enzymatic action or pH alteration. To explain, colon-specific ibuprofen delivery can be used to demonstrate a controlled-rate delivery method derived from cross-linked guar gum hydrogel functioning as a cross-linker with glutaraldehyde [88]. Todor et al., 2022, show that starch hydrogels can be considered perfect choices for target drug delivery in the colon. This is because not only do starch molecules have potential to become modulated to manage pharmacokinetics in the colon, but they also can be disgraced by commensal bacteria that causes the creation of SCFAs and many others as examples of health-promoting metabolites [89]. To highlight this point, Suhail et al.’s study can be named as another example that has proved that CS/β-CDcPAa hydrogels via different functions in pH 1.3 in comparison with 7.4, managed by the volume of CS/β-CDc polymer as a pH-sensitive network, can be a good choice for colon-targeted therapy [90].

### 5.4. Hydrogels for Sustained Delivery of Proteins and DNA

Biocompatible and hydrophilic are two main characteristics of hydrogels that make them ideal options for sustained DDS of proteins and DNA that leads to an increase in patient compliance. For example, Wei et al. concluded that interleukins are now delivered through hydrogels via injection methods to release interleukins as a kind of protein. In this research, Wei et al. used a physically cross-linked DNA hydrogel as a scaffold for delivery of interleukin-10 in diabetic conditions. This is because this method helps to release interleukin-10 continuously for a long time, which causes improvement in diabetic alveolar bone rebuilding via boosting osteogenesis as well as M2 phenotype polarization [62].

### 5.5. Hydrogels in Cancer Therapy

It is evident that cancer, as an aggressive disease with a high mortality rate, has negative effects on patients’ quality of life as they cope with this hostile disease, which results from treatments not having impacts on target sites and causing many harsh side effects. Consequently, creating a delivery system to handle and fix such problems can have many advantages over other delivery systems for treating cancers. To achieve this goal, Wang et al. worked on this problem, which led to thermos-responsive hydrogel-based on chitosan–poly (N-isopropyl acrylamide-co-acrylamide) as a temperature-dependent delivery system that is able to deliver cancer drugs via cellular uptake to fight against tumor hyperthermia conditions [81]. Moreover, according to Yi-Jun Jo et al., it has been indicated that DOX-loaded hydrogels not only showed anti-tumor activity as much as free-DOX but also that the combination of DOX + ICG-loaded hydrogels can be more efficient in order to fight against tumor cells [91].

### 5.6. Applications of Hydrogels in Gynecology

**Figure 4:**
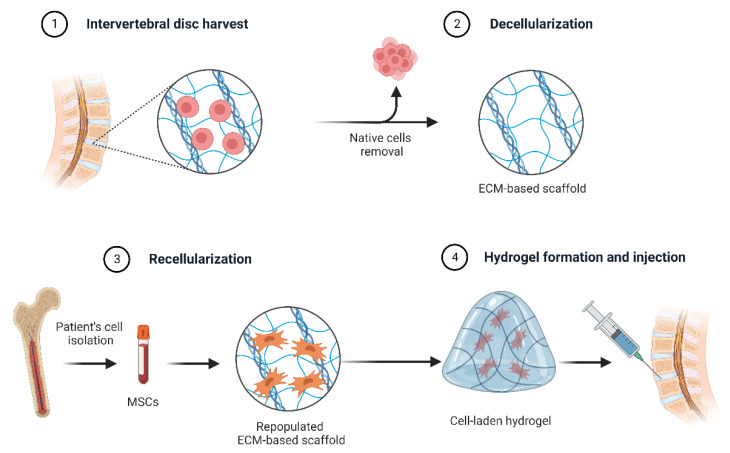
Strategies for regenerating the intervertebral disc using extracellular matrix. Decellularized tissue-based extracellular matrix (ECM) scaffolds have been studied widely in regenerative medicine for use in tissue regeneration.

Vaginal administration of drugs reaches some goals: not only for contraception or treating vaginal infections but also for improving the health conditions of the vaginal canal, both of which have been challenging for researchers. Having a suitable DDS for the vaginal canal to fight against vaginal cancer or contraceptive delivery has been a challenge in medical science. Polysaccharide-based hydrogels have gained attention in this field through hydrogel-based intravaginal devices because of having priorities over other delivery systems, including high drug loading, tunable release rate, ease of application, and becoming retained in situ [92]. From the research performed previously, it has been concluded that poloxamer hydrogel can be a good choice because it remains in the form of gel at room temperature. However, it becomes liquid after reaching its target site in the vaginal canal. Moreover, deformable propylene glycol-containing liposomal-based hydrogels are another kind of delivery system arising from hydrogels to fight against vaginal microbial infections via releasing anti-microbial drugs under the control of release rate. Clotrimazole, an anti-fungal drug used in the form of hydrogel to manage vaginitis, can be considered a perfectly matched example in this field to clarify the importance of hydrogels for the vaginal DDS [93]. Regarding this topic, Buckenmeyer et al. studied premature ovarian failure via extracellular matrix (ECM) ovarian hydrogels as a follicle carrier, which is schematically represented in Figure 4 [94,95]. This figure represents the strategies for regenerating the intervertebral disc using extracellular matrix and decellularized tissue-based extracellular matrix (ECM). Additionally, Hou et al. used 3D vaginal matrix bioink to encapsulate bone marrow mesenchymal stem cells (BMSCs) that led to a biocompatible 3D scaffold, which delivered nutrition and oxygen to the cells and allowed them to live on for a long time [96].

### 5.7. Hydrogels for Buccal Delivery

The buccal delivery system has always been regarded a perfect method for drug administration. This idea derives from having advantages over other systems including the ease of use leading to the improved compliance of patients, limited side effects, and rapid onset of action. In addition, it can be removed extremely quickly in an emergency against other routes of administration. Higher permeability (which means the amount of water flowing within a polymer layer when it is swollen) is another beneficial property of the buccal route that comes from having a lot of blood vessels in the buccal mucosa. To be more specific, chitosan, hydroxypropyl cellulose (HPC), hydroxypropyl methylcellulose (HPMC), carboxymethyl cellulose (CMC), and polyacrylic (PA) resins-based hydrogels are some famous examples of hydrogels for buccal delivery system. Hydrogels’ form of poly (methacrylamide–co-N-vinyl-2-pyrrolidone co-itaconic acid) is a special sort of hydrogel that achieves the aforementioned aim. Gengigel^®^ (Oraldent Ltd., Cambridgeshire, UK) is a hyaluronan-based mouth and gum care product that is commercially available and has been used for mouth ulcers. Notably, the release of insulin can be controlled by using hydrogels in responding to the glucose level in patients’ blood. To clarify, Ghosh et al., 2020, showed that bio adhesive hydrogels may be considered perfect choices for buccal drug delivery since they are able to stick to the mucosal tissue in the buccal area and release their drugs gradually. Isosorbide mononitrate for managing chest pain in angina patients, Benzocaine as an anesthetic to control pain in the oral area, and Prochlorperazine maleate for treating nausea and vomiting are some of the best examples of hydrogels delivered by buccal route mentioned in this. Moreover, Cascone et al., via gathering information around this topic, professed that Xanthan gum and carbomer are two famous examples of hydrogels that have been utilized for buccal drug delivery, stemming from their characteristics, such as high ability to spread, wetting, swelling, viscoelasticity, perfect adhesion to the buccal mucosa, and bio-adhesive properties, not only in dry but also in liquid states, along with low cost, biodegradability, and non-toxicity, which make them appropriate options in this field [97]. Table 1. represents the hydrogel-based products in the biomedical market.

## 6. Conclusions and Perspective

It has been proved that polysaccharide-based hydrogel hydrophilic polymeric networks have many advantages over other DDSs used in health-oriented and cosmetic fields. Polysaccharide-based hydrogels have tremendous potential for medicinal applications, including tissue engineering and controlled drug release for targeted therapy. The most remarkable feature of these materials is the capacity to be injected without changing their chemical, mechanical, or physiological characteristics, which is accomplished by utilizing their thixotropic nature. Polysaccharide-based hydrogels can alter their rheological and chemical–physical properties by adjusting the cross-linking agents and taking advantage of their thixotropic tendency. Hydrophilic polymer chains interact physically or chemically to form hydrogels with a high water-absorption capacity. The hydrogel resembles biological tissues because water molecules can penetrate the three-dimensional polymeric network’s interstitial areas. As mentioned earlier, biomedical applications including drug delivery, wound healing, tissue engineering, and sustained delivery of proteins and DNA are some examples of hydrogels’ benefits, derived from characteristics of hydrogels such as biocompatibility, nontoxicity, degradability, swelling properties, light stability, and so many others. Since each hydrogel made of a particular polysaccharide must be researched separately, no behavior applies to all polysaccharide-based hydrogels. Future research on hydrogels can include (i) the effects of hydrogels, which should be investigated for long-term uses to ensure their safe clinical use based on evidence of dealing with human bodies via in vivo studies to limit their side effects and (ii) attempts to increase the stability of all kinds of hydrogels during storage time.

## Figures and Tables

**Table 1:** Represents the hydrogel-based products in the biomedical market.

No	Category	Product Name	Agent	Medical Application	Type/Form of Hydrogel	Polymer Name	Reference
**1**	Wound dressings	Suprasorb^®^ G	*	Adjusting moisture and the pain of the wound, removing necrotic tissue, relieving pain via cooling and soothing effect	Hydrogel film	Acrylic polymers, polyethylene, and phenoxyethanol with 70% water content	[98]
**2**	Wound dressings	AquaDerm™	2-Acrylamido-2 methyl-1 propanesulfonic acid sodium	Pressure ulcers, minor burns, radiation tissue damage	Hydrogel sheet	Propylene Glycol, poly (ethylene glycol) dimethacrylate, 2-Hydroxy-2-methylpropiophenone with 38–55% water	[99]
**3**	Wound dressings	SOLOSITE^®^ Gel	*	With creation of a moist wound environment: Minor burns Superficial lacerations Cuts and abrasions (partial thickness wounds) Skin tears with the creation of a moist wound environment Venous ulcers (leg ulcers) Surgical incisions Diabetic foot ulcers Pressure ulcers (including stage IV) Assistance in autolytic debridement of wounds covered with necrotic tissues	Gel form of hydrogels	Sodium salt of carboxymethyl cellulose and glycerol with above 60% water	[100]
**4**	Cosmetic products	Rose soothing hydrogel mask	*	Face skin (it soothes and rejuvenates the skin)	Face mask	Sodium polyacrylate, glycerin, cellulose gum, water, etc	[101]
**5**	Cosmetic products	Advanced Genifique hydrogel Mask	*	Face skin (skin will become moisturized, radiant, smoother)	Face mask	Water, etc., enriched with Bifidus extract	[102]
**6**	Contact lenses	Focus^®^ DAILIES^®^ with AquaRelease™	*	Astigmatism	Lubricates the eye for every blink	*	[103]
**7**	Contact lenses	Airsoft™	*	Astigmatism	High water content and oxygen permeability	Silicone hydrogel material	[104]
**8**	Cartilage	*	*	Chondrogenesis	Visible pores, 3D structural integrity, good biocompatibility and proliferative activity	Biotinylated-HA, sodium alginate	[105]
**9**	Cartilage	*	TGF-β1	Cartilage regeneration	3D bioprinting HA-based hydrogels in tissues engineering	Thiol-modified HA	[106]
**10**	Bone	*	Maleimide	tissue engineered bone substitutes	3D bioprinting HA-based hydrogels	HA	[107]
**11**	Vascular	*	*	Angiogenesis	3D bioprinting HA-based hydrogels	HA glycidyl methacrylate, polylactic-co-glycolic acid (PLGA)	[108]
**12**	Vascular	*	collagen-I	Boosting neuronal development, improving peripheral nerve regeneration	3D bioprinting HA-based hydrogels	Methacrylated HA	[109]
**13**	Nervous	*	Dopamine-conjugated HA, dopamine-conjugated gelatin, thiolated Pluronic F-127	Nerve tissue regeneration	3D bioprinting HA-based hydrogels	HA, conjugated gelatin	[110]
**14**	Cardiac	*	*	Cardiac tissue engineering	3D bioprinting HA-based hydrogels	Methacrylated HA, methacrylated gelatin	[111]
**15**	Wound dressings	*	*	Dressing to enhance skin wound healing	Temperature- responsive hydrogels	Methylacrylate gelatin	[112]
**16**	Tissue engineering	*	*	Neural tissue engineering	Light/photo- responsive hydrogel	Conducting polymer hydrogel (CPH) based on copolymerized PANI and PAM (PAM/PANI CPH)	[113]
**17**	Drug delivery	*	*	Controlled drug delivery	PH-responsive hydrogels	Poly(methacryloyloxyethyl phosphorylcholine-co-4- formylbenzoate ethyl methacrylate) P(MPC-co-FBEMA) copolym	[114]
**18**	Drug delivery	*	*	Drug Delivery	Glucose-responsive hydrogels	Phenylboronic acid-grafted γ-Polyglutamic acid (PBA-PGA)	[115]
**19**	Corneal tissue engineering	*	Adrenaline and Chloramphenicol	Ocular drug delivery system	Temperature- responsive	Gellan maleate (MA-G)	[116]
**20**	Osseous tissue engineering	*	NaF, BSA, and BMP-2	Cargo for delivery of different therapeutics	Temperature and Ultrasound- responsive	Alginate (Alg.)	[117]
**21**	Tendinous tissue engineering	*	PL	Delivery of PL for release of PL-derived growth factors	Magnetic- Responsive	Methacrylated chondroitin sulfate (MA- CS)	[118]
**22**	Meniscal tissue engineering	*	TGF-β1	Release of TGF-β1 to manage the fibrochondrogenic differentiation of BMSCs and develop meniscal defects in rabbit model	Temperature- responsive	Glycol Chitosan GC/4- Arm PEG-CHO Hydrogel	[119]

* not determined

## Data Availability

The underpinning data can be publicly accessed via DOI: 10.25383/city.21706886.
